# Caerin1.1 Suppresses the Growth of Porcine Epidemic Diarrhea Virus In Vitro via Direct Binding to the Virus

**DOI:** 10.3390/v10090507

**Published:** 2018-09-18

**Authors:** Nan Guo, Bingzhou Zhang, Han Hu, Shiyi Ye, Fangzhou Chen, Zhonghua Li, Pin Chen, Chunmei Wang, Qigai He

**Affiliations:** 1 Division of Animal Infectious Diseases, State Key Laboratory of Agricultural Microbiology, College of Animal Sciences and Veterinary Medicine, Huazhong Agricultural University, Wuhan 430070, China; guonan0609@163.com (N.G.); abing0313@webmail.hzau.edu.cn (B.Z.); huhanhzau@outlook.com (H.H.); yeshiyi@webmail.hzau.edu.cn (S.Y.); chenfangzhou@webmail.hzau.edu.cn (F.C.); lzh1990@webmail.hzau.edu.cn (Z.L.); chenpin@mail.hzau.edu.cn (P.C.); 2 Key Laboratory of Veterinary Chemical Drugs and Pharmaceutics, Ministry of Agriculture, Rural Affairs, Shanghai Veterinary Research Institute, Chinese Academy of Agricultural Sciences, Shanghai 200241, China; wangchunmei@shvri.ac.cn

**Keywords:** antimicrobial peptides, Caerin1.1, porcine epidemic diarrhea virus

## Abstract

Porcine epidemic diarrhea (PED) has re-emerged in recent years and has already caused huge economic losses to the porcine industry all over the world. Therefore, it is urgent for us to find out efficient ways to prevent and control this disease. In this study, the antiviral activity of a cationic amphibian antimicrobial peptide Caerin1.1 against porcine epidemic diarrhea virus (PEDV) was evaluated by an in vitro system using Vero cells. We found that even at a very low concentration, Caerin1.1 has the ability to destroy the integrity of the virus particles to block the release of the viruses, resulting in a considerable decrease in PEDV infections. In addition, Caerin1.1 showed powerful antiviral activity without interfering with the binding progress between PEDV and the receptor of the cells, therefore, it could be used as a potential antiviral drug or as a microbicide compound for prevention and control of PEDV.

## 1. Introduction

Porcine epidemic diarrhea virus (PEDV) is an enveloped virus that belongs to the genus *Alphacoronavirus* of the family *Coronaviridae* with single-stranded positive-sense RNA [1]. It is the causative agent of an acute infectious enteric disease known as porcine epidemic diarrhea (PED) that is clinically manifested by severe watery diarrhea, vomiting, and dehydration in the suckling piglets [2]. With the high mortality in the piglets, PED infection finally caused enormous economic losses to the global swine industry, especially after its recent re-emergence caused by variant PEDV strain throughout the world [3,4]. However, the classical PED vaccines could not provide appropriate protection against the variant PEDV infection. Considering this, relevant studies of new antiviral materials are needed to prevent and control emerging or re-emerging infectious diseases such as PED [5].

Antimicrobial peptides (AMPs) are important components of the nonspecific immune system of animals to eradicate invaders [6], and the skin secretion of anuran amphibians are rich sources for collecting AMPs [7,8,9]. AMPs have a wide spectrum of antimicrobial activity against microorganisms such as bacteria, viruses, fungi, and parasites [10,11], but are very friendly to host cells [12,13,14]. Moreover, AMPs can work as growth and health promoters to improve the performance of pigs by enhancing the immune status, improving the intestinal health, and alleviating the toxic effects of deoxynivalenol in pigs [15]. There are also research findings that AMPs can modulate immune responses like chemokines, cytokine production, and pro-inflammatory responses [16,17].

Caerin1.1 is a peptide from the granular glands within the skin of the Australian green tree frog with 25-residues (GLLSV LGSVA KHVLP HVVPV IAEHLNH2). Nuclear magnetic resonance (NMR) of Caerin1.1 in the membrane mimetic environments showed that it has two α-helices and a flexible hinge region composed of two prolines [18]. Both prolines are essential for the antimicrobial activities [19]. Some studies have reported that Caerin1.1 exhibits antibacterial and antiviral properties by forming pores on the membrane to destroy the integrity of the particles [20,21]. Meanwhile, it has been confirmed that Caerin1.1 has a complete inhibitory effect against HIV by preventing viral fusion to target cells and disrupting the HIV envelope, and, remarkably, that Caerin1.1 is also highly effective in inhibiting the transfer of HIV from dendritic cells (DCs) to T cells even when DCs are continuously exposed to peptides for 8 h after virus capture, and that Caerin1.1 has a bacteriostatic activity against *Escherichia coli* and *Bacillus subtilis* [8,21]. Although the mechanism that suppresses the activities of some bacteria and viruses has been illustrated clearly, it remains unknown whether Caerin1.1 can inhibit the growth of PEDV. So, this study is aimed to investigate the function of Caerin1.1.

For this purpose, we investigated the inhibitory ability and antiviral mechanisms of Caerin1.1 against different PEDV strains in Vero cells, which will provide an insight into AMPs’ antiviral mechanisms and its application as antiviral drugs or as drug loading compounds.

## 2. Materials and Methods

### 2.1. Cells, Viruses and Antimicrobial Peptides

Vero cells (African green monkey kidney cell lines) were propagated at 37 °C in a 5% CO_2_ humidified incubator using Dulbecco’s Modified Eagle Medium ((DMEM), Gibco, Langley, VA, USA) containing 10% fetal bovine serum (Invitrogen, Carlsbad, CA, USA). Cells were infected with three different PEDV strains: YN (Accession No. KF761675), CV777 (Accession No. KT323979), and DR13 (Accession No. JQ023161). The YN strain CH/YNKM-8/2013 was isolated from a suckling piglet suffering from acute diarrhea. The cells infected with PEDV strains were cultured in DMEM supplemented with 10 μg/mL trypsin.

Caerin1.1 and N-terminus FITC labeled Caerin1.1 (purity: both >95%) were chemically synthesized by Bioyeargene (Wuhan, China). Caerin1.1 was initially dissolved in 0.01% acetic acid to reach the concentrations of 1 mg/mL and 5 mg/mL as stock solutions, respectively and stored at −80 °C until further use [22].

### 2.2. Cytotoxicity Assay

The cytotoxicity of Caerin1.1 in Vero cells grown in 96-well culture plates was assessed by MTT (3-(4,5-dimethyl-2-yl)-2,5-diphenyltetrazolium bromide, 5 mg/mL) assay. The cells were incubated with Caerin1.1 at different concentrations for 72 h, then 20 μL/well MTT was added and cultured for additional 4 h. The supernatant was then removed and dimethylsulfoxide (DMSO) was added to culture plates (150 μL per well), then the plates were shaken at room temperature (RT) for 10 min. Finally, the optical density (OD) value was measured at the wavelength of 490 nm [23].

### 2.3. Plaque Forming Assay

The plaque forming assay was performed on Vero cells cultured in 12-well plates. PEDV (200 pfu/well) was incubated using different concentrations of Caerin1.1 for 1 h at 37 °C before infection and then incubated PEDV was added on Vero cells covering 80% monolayer. After 1 h of viral adsorption, the supernatant was removed, and the cells were rinsed with PBS and overlaid with 10 μg/mL trypsin supplemented with sodium carboxymethyl cellulose-containing medium. The plates were fixed with 10% formaldehyde after 72 h infection, and then stained with crystal violet solution [24].

### 2.4. Immunofluorescence Assays (IFA)

Vero cells cultured in 24-well plates were washed with PBS for 3 times and inoculated respectively with single medium, or single PEDV, or PEDV pre-incubated with different concentrations of Caerin1.1. The cells were rinsed with PBS after 24 h infection, the cells were fixed with 4% formaldehyde for 15 min at RT. Then the cells were incubated with an anti-PEDV monoclonal antibody (made in our laboratory) for 1 h and fluorescein isothiocyanate (FITC)-conjugated goat anti-mouse antibody (1:60 dilution) for 1 h at 37 °C. The immunofluorescence images were taken with a Nikon Eclipse Ti microscope (Nikon, Tokyo, Japan) [25].

### 2.5. Western Blot Analysis

Vero cells were cultured in 6-well plates and infected with PEDV, and treated with Caerin1.1 at different time points. The cells were rinsed three times with PBS at 20 h post infection (hpi), and treated with 120 μL/well lysis solution containing protease inhibitors (PMSF). Then 30 μL of sodium dodecyl sulfate (SDS) loading buffer was added to the cell extracts and the samples were boiled for 10min. The proteins were separated by 12% sodium dodecyl sulfatepolyacrylamide gel electrophoresis (SDS-PAGE) and then transferred into PVDF membranes (Millipore, Mississauga, ON, Canada). Membranes were blocked with 5% skimmed milk for 2 h at 37 °C and then incubated with primary antibodies over night at 4 °C. The blots were then incubated with corresponding horseradish peroxidase (HRP)-conjugated secondary antibodies (ABclonal, Wuhan, China). The membranes were washed 3 times at each step. The protein bands were visualized using the Clarity™ Western ECL Blotting Substrate (Bio-Rad, Hercules, CA, USA). The protein blots were quantified by Image J software (National Institutes of Health, Bethesda, MD, USA).

### 2.6. Transmission Electron Microscopy Observation

PEDV-infected cell culture was prepared and centrifuged at 4 °C for 30 min at 2770× *g* to get rid of the cell debris. The supernatants were ultracentrifuged at 4 °C for 2 h at 200,500× *g*. Then the purified virus pellets were re-suspended with PBS and treated with Caerin1.1 (10 μg/mL) for 1 h at 37 °C and the viruses treated in the same manner were used as positive control. Afterwards, the cocktail was dripped on the copper grid and negatively stained with phosphotungstic acid (PTA). Finally, the samples were examined using the electron microscope (Hitachi H7500, Tokyo, Japan) [26].

### 2.7. Assays of Antiviral Activity

#### 2.7.1. Viral Inhibition Assays

PEDV suspensions containing different concentrations of Caerin1.1 were pre-incubated for 1 h at 37 °C, and were serially diluted before they were inoculated on the 80% confluent Vero cell monolayers grown in the 96-well plates, followed by washing 3 times with PBS. About 1hpi, the inoculates were removed and the cells were washed again with PBS for 3 times and incubated for another 3 days with DMEM containing trypsin (10 μg/mL). The infectivity was calculated by TCID_50_ (Tissue Culture Infectious Dose 50) following the Reed-Muench method established by L. J. Reed and H. Muench [27].

#### 2.7.2. Infectious Virus Yield Reduction Assay

The Vero cell monolayers were infected with PEDV (200 pfu). At 1 hpi, the cells were washed twice with PBS and then treated with different concentrations of Caerin1.1, and at 12 hpi, the culture supernatants and the cells were collected respectively [28]. The cell samples were frozen and thawed 3 times to completely release the intracellular virus particles. Both the extracellular and intracellular virus yields were determined by TCID_50_.

#### 2.7.3. Time of Addition Assay by TCID_50_

The Vero cells in 12-well plates were inoculated with PEDV in the presence or in the absence of Caerin1.1 at different time points: a. 1 h before infection (pre); b. incubation of Caerin1.1 and PEDV for 1 h before infection (co); c. 1 hpi (post). The supernatants and the cells were collected together at different time points and the samples were treated in the same way mentioned above in an infectious virus yield reduction assay. On the other hand, the inhibitory ability of Caerin1.1 against PEDV was examined using a 10-fold higher concentration for further understanding antiviral activities in the pre and post treatment situations. Finally, the cell lysates were collected in a parallel set for western blot assay.

Cells were pre-chilled at 4 °C for 1 h before infection and were chilled again after inoculated with PEDV for 3 h, then the cells were washed with ice cold PBS 3 times, replenished with DMEM containing trypsin, were incubated at 37 °C for 72 h, and the inhibitory effects were determined by TCID_50_. Caerin1.1 was separately added at two time points: together with PEDV at the time of viral absorption, and after the cells were washed at the time of viral entry to reach a highest final concentration of 10 μg/mL. The cell lysates were processed in the same procedures with western blot analysis [29].

### 2.8. Confocal Laser Scanning Microscopy

Vero cells were seeded onto some glass coverslips and were separately treated with FITC-Caerin1.1 (100 μg/mL), PEDV (200 pfu), and both FITC-caerin1.1 (100 μg/mL) and PEDV (200 pfu), then incubated at 37 °C for 12 h. Cells were fixed with 4% formaldehyde followed by permeabilization for 30 min with 0.1% Triton X-100, and were blocked with 1% BSA for 1 h. The cells were washed 3 times with PBS at each step. Cell nuclei were counterstained with 0.01% 4′,6-diamidino-2-phenylindole ((DAPI), Invitrogen), and the antibodies used here were: PEDV-S monoclonal antibody (made in our laboratory) and Alexa Fluor 594-conjugated Affinity Pure Donkey Anti-Mouse IgG (H+L) (Ant Gene, Wuhan, China). The samples were examined using a confocal microscope (LSM 510 Meta, Carl Zeiss, Munich, Germany) [30].

### 2.9. Statistics

All experiments were performed with three independent experiments, and the calculated results were presented as mean ± standard deviation (SD). Statistical analyses were performed using student’s *t*-test. Graph Pad Prism5.0 was used to analyze the statistics in this study. The statistical significances were defined as *p* < 0.05 (*), and the higher significance was denoted by *p* < 0.01 (**) and *p* < 0.001 (***).

## 3. Results

### 3.1. Cytotoxicity of Caerin1.1

**Figure 1:**
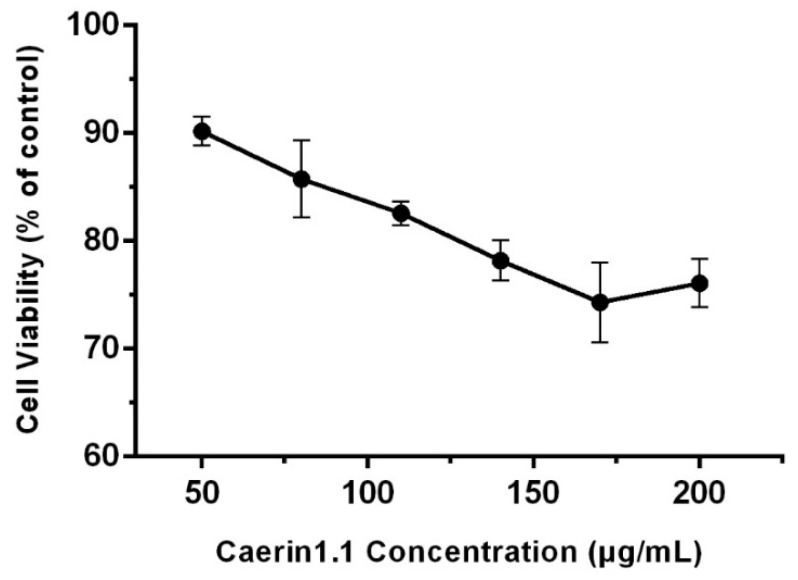
The cytotoxicity of Caerin1.1 on Vero cells. Vero Cell viability was measured by MTT. All the MTT values were normalized based on the control (with no Caerin1.1) which represents 100% cell viability.

The result of cytotoxicity assay indicated that cell viability increased with the decrease of the concentration of Caerin1.1, that the viability of cells remained over 80% when treated with Caerin1.1 at the concentrations not exceeding 110 μg/mL, and that the cell morphology was not affected even at the concentration 140 μg/mL with a cell viability value of 76%, as seen in Figure 1. Thus, 10 μg/mL Caerin1.1 was chosen as the highest final concentration in most of the subsequent experiments. We used 20 μg/mL, 10 μg/mL, 5 μg/mL, and 2.5 μg/mL of Caerin1.1 in the IFA assay and in the addition assay.

### 3.2. Optimization of Suitable Incubation Condition of PEDV and Caerin1.1

**Figure 2:**
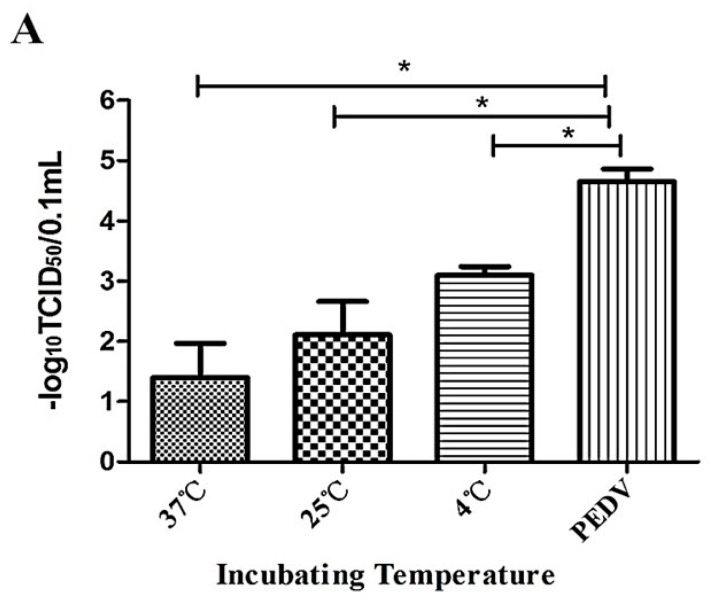

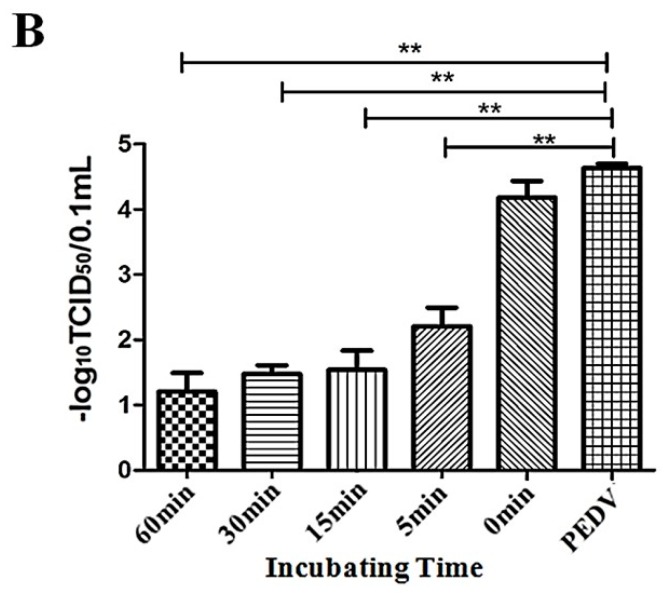
The most suitable incubating conditions of porcine epidemic diarrhea virus (PEDV) and Caerin1.1. The titer of PEDV was detected under different conditions. (**A**) Incubating PEDV with Caerin1.1 (10 μg/mL) at different temperatures (37, 25, 4 °C) for one-hour pre-infection; (**B**) Incubating PEDV with Caerin1.1 (10 μg/mL) at different time points (60, 30, 15, 5, 0 min) at 37 °C pre-infection. The control groups (PEDV) of A and B were treated without any Caerin1.1 at 37 °C. The results are presented as the mean ± SD of three independent experiments. The statistical analysis was performed using Graph Pad Prism5.0. Significance was defined as *p* < 0.05 (*), and higher significance was defined as *p* < 0.01(**).

Different incubating conditions of PEDV in the presence of Caerin1.1 can directly affect the antiviral activity of Caerin1.1. In this experiment, we determined the titer of PEDV under different incubating temperatures and time durations. The results showed that the most suitable incubating temperature was 37 °C, shown in Figure 2A, and time length was 1 h, shown in Figure 2B. Low temperature and inadequate incubation time would obviously affect the inhibition activity of Caerin1.1 against PEDV.

### 3.3. The Inhibitory Effect of Caerin1.1 against PEDV-YN Strain

To fully understand the inhibitory effect of Caerin1.1 against PEDV-YN strain, several experiments were performed including cytopathic effects (CPE) observation, plaque reduction assay, TCID_50_, and IFA.

**Figure 3:**
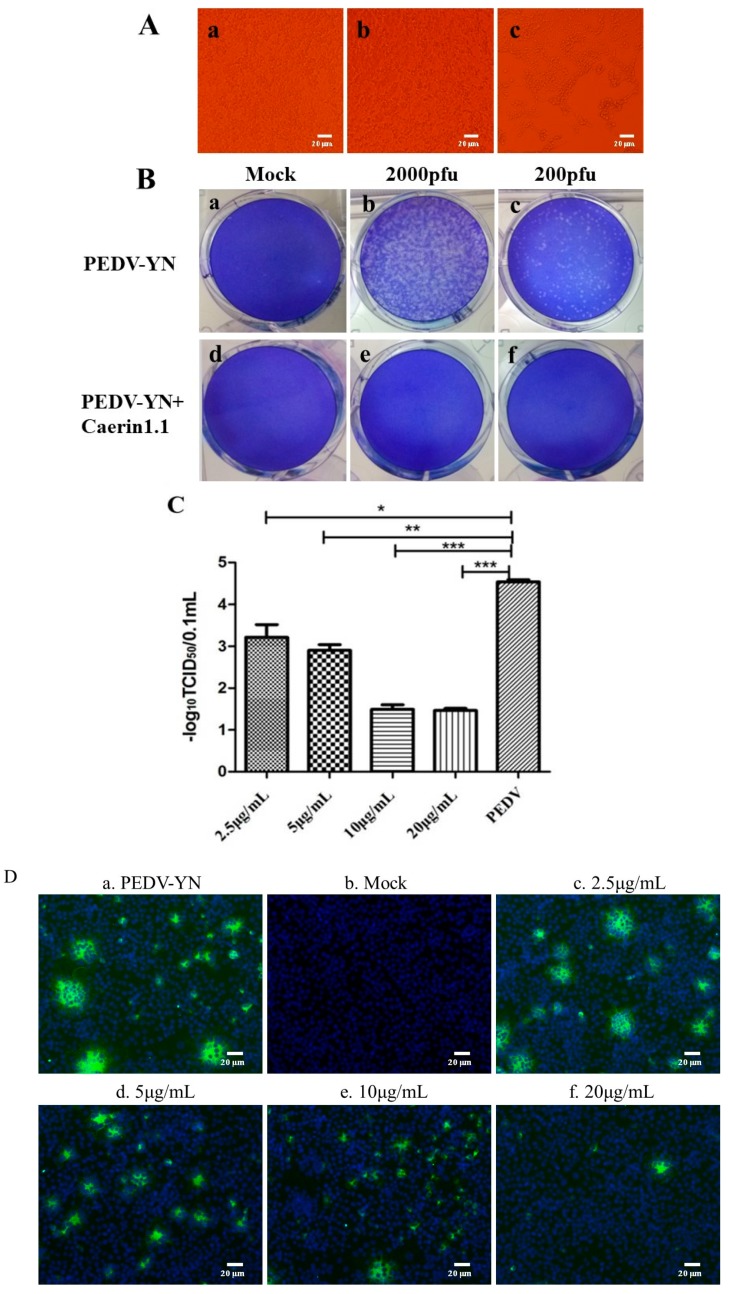
The inhibitory effect of Caerin1.1 against porcine epidemic diarrhea virus (PEDV)-YN strain. (**A**) (a) mock-infected cells, PEDV-infected Vero cell morphology in the presence (10 μg/mL) (b) or absence (c) of Caerin1.1; (**B**) The number of plaques (clear spots) observed represents the number of viruses in a given dilution (a) Mock-infected cells; Cells infected with PEDV titer of 2000 pfu (b) and 200 pfu (c); (d) cells treated with 10 μg/mL Caerin1.1; Cells infected with PEDV titer of 2000 pfu (e) and 200 pfu (f) in the presence of Caerin1.1 (10 μg/mL); (**C**) The titer of PEDV was detected under the treatment of different concentration of Caerin1.1 (2.5, 5, 10, 20 μg/mL) after one hour. The control groups (PEDV) were not treated by Caerin1.1. The results of three independent experiments are presented as the mean ± SD. The statistical analysis was performed using Graph Pad Prism5.0. The significance was defined as *p* < 0.05 (*), and the higher significance was defined as *p* < 0.01(**), *p* < 0.001(***); (**D**) Fluorescence intensity of PEDV in the presence of Caerin1.1 at different concentration. (a) Cells infected with PEDV of 200 pfu; (b) Mock-infected cells; PEDV were incubated with Caerin1.1 of 2.5 (c), 5 (d), 10 (e) and 20 (f) μg/mL for 1 h. Scale bar, 20 µm.

The CPE observation results showed that there were remarkable differences between the cells infected with PEDV in the absence or presence of Caerin1.1. Caerin1.1-treated cells were in a relatively normal status, and the CPE appeared in Caerin1.1-treated group about 30 h later than in the PEDV control group, as seen in Figure 3A. With the addition of Caerin1.1, the number of plaques declined at a surprising speed and the diameters decreased notably. Similar results can be observed even with 2000 pfu of PEDV treated by Caerin1.1. The cells infected with 200 pfu PEDV exactly remained the same as the cells in negative control group, as seen in Figure 3B.

Caerin1.1 significantly inhibited the multiplication of PEDV in a dose-dependent manner, demonstrating that PEDV infection to Vero cells was blocked to a large extent. The results of TCID_50_, shown in Figure 3C, and IFA, in Figure 3D, indicated that the titer and the fluorescence intensity of PEDV decreased significantly with the increase in the concentration of Caerin1.1.

### 3.4. The Inhibitory Effect of Caerin1.1 against Another Two PEDV Strains

**Figure 4:**
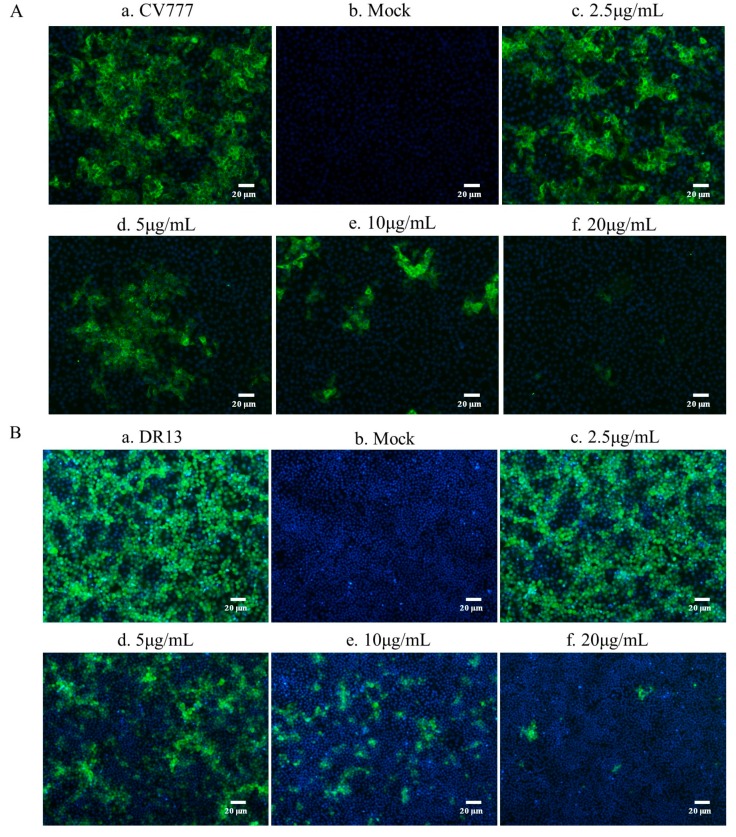
The inhibitory effect of Caerin1.1 against CV777 (**A**) and DR13 (**B**) strains. Fluorescence intensity of CV777/DR13 strain in the presence of Caerin1.1 at different concentrations. (a) Cells infected with porcine epidemic diarrhea virus (PEDV) of 200 pfu; (b) Mock-infected cells; PEDV were incubated with Caerin1.1 at the concentrations of 2.5 (c), 5 (d), 10 (e) and 20 (f) μg/mL for 1 h. Scale bar, 20 µm.

The inhibitory effects of Caerin1.1 against another two different PEDV strains were examined to evaluate the anti-PEDV potential of Caerin1.1 based on IFA. As shown in Figure 4, Vero cells were infected with PEDV (200 pfu) pre-incubated with different concentrations of Caerin1.1. Compared with the virus control, treatment group exhibited excellent inhibitory effects in a dose dependent manner even at extremely low concentrations of Caerin1.1. These results revealed that the intracellular viruses evidently reduced in the presence of Caerin1.1 in a dose dependent manner.

### 3.5. The Suppression of Virus Propagation

**Figure 5:**
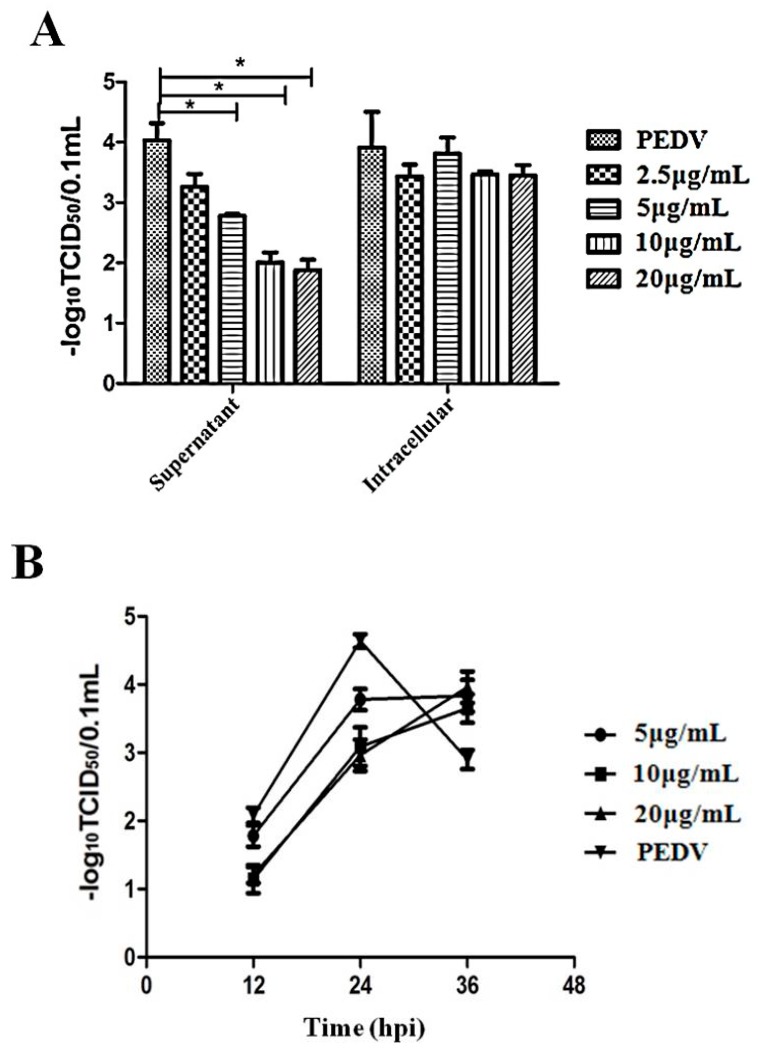
The inhibitory effect of Caerin1.1 against the progeny virus production of porcine epidemic diarrhea virus (PEDV). (**A**) The intracellular and extracellular progeny virus titers were detected after treatment with different concentration of Caerin1.1. The concentration of Caerin1.1 used in the experiments are 2.5, 5, 10, 20 μg/mL, respectively; (**B**) The progeny virus (extracellular virus) titers were also detected under treatment with different concentration of Caerin1.1 (5, 10, 20 μg/mL) at different time points (12, 24, 36 h). The results of three independent experiments are presented as the mean ± SD. The statistical analysis was performed using Graph Pad Prism5.0. The significance was defined as *p* < 0.05 (*).

To better understand the inhibitory effects of Caerin1.1 against the progeny virus production of PEDV, we added Caerin1.1 at 1 hpi to PEDV infected cells. And both the intracellular and extracellular virus titers were determined separately by using TCID_50,_ as shown in Figure 5A. The progeny virus titer decreased significantly as compared to that of virus control in a dose dependent manner, as seen in Figure 5A. On the other hand, extracellular virus titers were also determined at different time points and different Caerin1.1 concentrations. The results showed that the increase of extracellular virus titer slowed down under the treatment of Caerin1.1, as seen in Figure 5B.

**Figure 6:**
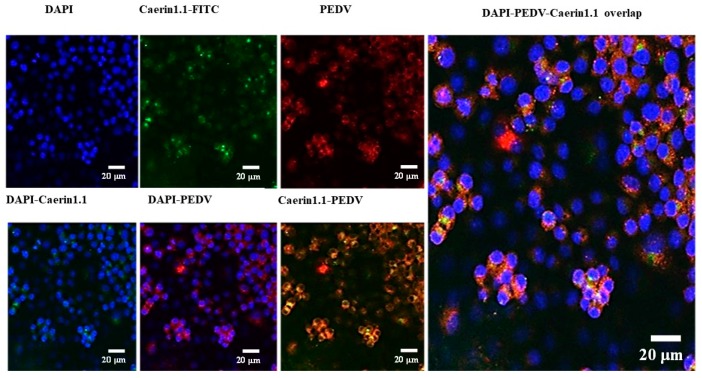
The interaction between Caerin1.1 and porcine epidemic diarrhea virus (PEDV). Confocal microscope. Vero cells were treated with fluorescein isothiocyanate (FITC)-Caerin1.1 (100 μg/mL), PEDV (200 pfu), or FITC-Caerin1.1 (100 μg/mL) and PEDV (200 pfu) separately, which were incubated with Vero cells for 12 h at 37 °C. Caerin1.1 were immunostained with FITC (green). The PEDV infection was detected with the monoclonal antibody against PEDV S protein (red) and cell nuclei were counterstained with 4′,6-diamidino-2-phenylindole (DAPI) (blue). Scale bar, 20 µm.

In the fluorescent confocal experiment, the fluorescence of Caerin1.1 and PEDV around cell nucleus could be observed when we incubated them with Vero cells respectively. Additionally, the distribution of Caerin1.1 and PEDV were almost the same, so the results of confocal laser scanning microscopy verified that Caerin1.1 could combine with PEDV, and that the antiviral function of Caerin1.1 in the cell plasma could be observed especially in the areas showing severe CPE, as seen in Figure 6.

### 3.6. Caerin1.1 Acts Mainly by Direct Inactivation of Viral Particles

**Figure 7:**
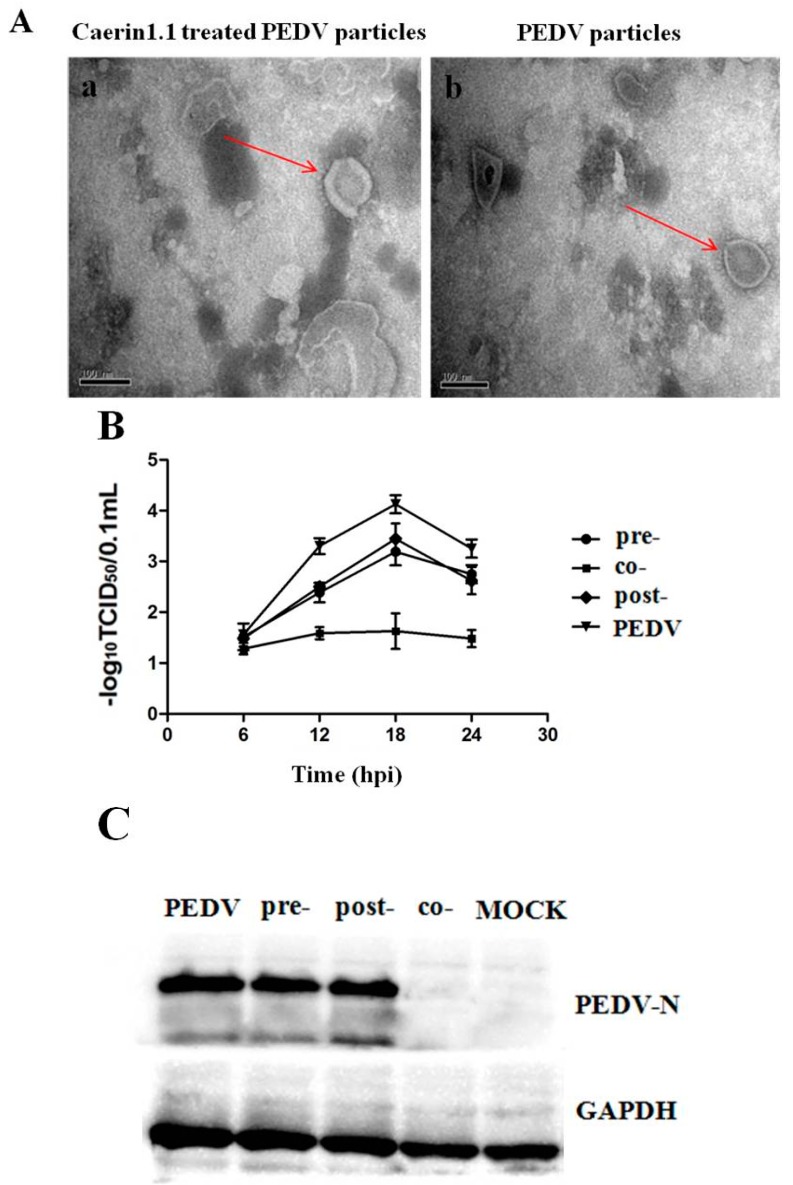

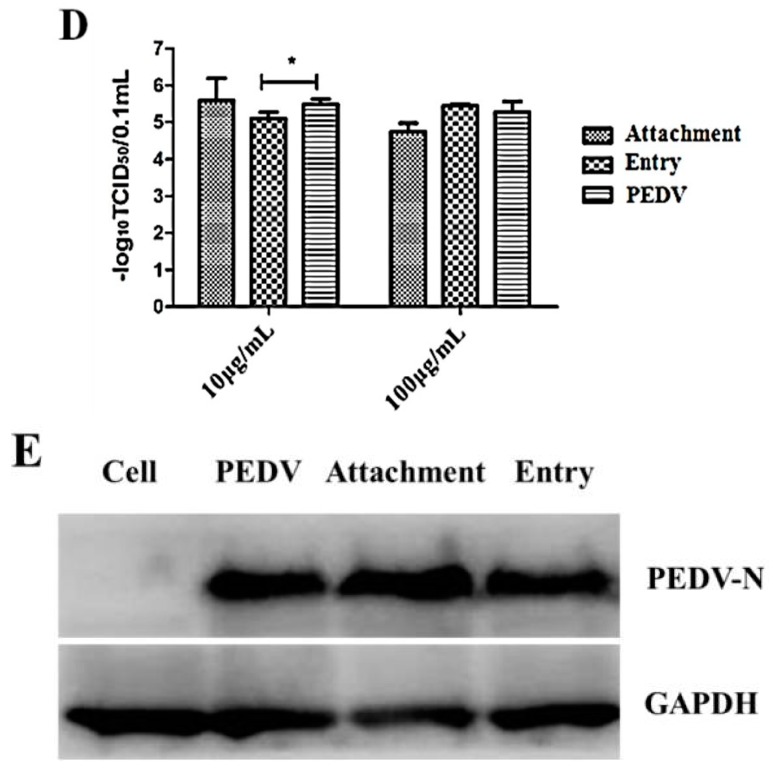
Caerin1.1 inactivates virus particles through direct interaction with porcine epidemic diarrhea virus (PEDV) particles. (**A**) Transmission electron microscopic images of Caerin1.1-treated viruses. (a) PEDV incubated with Caerin1.1 (10 μg/mL) for 1 h at 37 °C. (b) PEDV control. In the images, the arrows indicate the complete (b) or destructed PEDV particles (a). Scale bars: 100 nm; (**B**) Tissue Culture Infectious Dose 50 (TCID_50_) of Vero cells infected with PEDV under different concentration treatments of Caerin1.1. Pre-: Caerin1.1 was added 1 h before virus attachment. Co-: Caerin1.1 was incubated with PEDV for 1 h and the incubation compound was added to Vero cells. Post-: Caerin1.1 were added to Vero cells 1 h after virus entry process. PEDV without Caerin1.1 treatment acts as the control group; (**C**) Western blot analysis of the expression level of PEDV-N protein in different treatments of Caerin1.1 as shown in Figure 7B. GAPDH (glyceraldehyde-3-phosphate dehydrogenase) was used as a loading control; (**D**) TCID_50_ showed that Caerin1.1 had no obvious inhibitory effects during the attachment and entry process. Caerin1.1 was added at PEDV attachment and entry periods, then the titers of PEDV were detected; (**E**) Western blot analysis of the expression level of PEDV-N protein when Caerin1.1 was added in different ways as shown in Figure 7D. GAPDH was used as a loading control.

In Caerin1.1-PEDV pre-incubation process, the morphological changes of PEDV particles were observed clearly. So Caerin1.1 could destroy the structure of the virus to block the infection of the host cells, as seen in Figure 7A, resulting in the inhibition of viral release, which in turn could reduce the transmission of the progeny virus among the adjacent cells. As shown in the growth curve, PEDV co-incubated with Caerin1.1 showed the lowest titer, while PEDV was only slightly inhibited in pre-incubation process of Caerin1.1-cells and the delayed usage of Caerin1.1, as seen in Figure 7B. In a western blot experiment, the protein band could barely be observed in PEDV-Caerin1.1 co-incubation group. However, no obvious differences in PEDV-N protein expression were observed among the cell-Caerin1.1 pre-incubation group, the post treatment group, and PEDV control group, as seen in Figure 7C.

To further explore whether Caerin1.1 could bind to some virus receptors in host cells to interfere with the attachment and entry process, we kept the cells at 4 °C to maintain the attaching status. Caerin1.1 did not show obvious inhibitory effects during the attachment and entry process. There were no significant differences in virus titers between the virus control groups and Caerin1.1 treated groups during both attachment period and entry period, even if the concentration of Caerin1.1 was increased tenfold, as seen in Figure 7D. The western blot assay did not exhibit any obvious differences in protein expression among attachment period treated group, entry period treated group, and the PEDV control group, shown in Figure 7E, either.

## 4. Discussion

PEDV is one of the most important pathogens leading to diarrhea in pig industry. The aim of this study is to find whether Caerin1.1 has the antiviral activity against the three PEDV strains and to reveal the antiviral mechanism of Caerin1.1.

According to published data, Caerin1.1 is a cationic peptide with two α-helices and has proved to be an effective agent against HIV by damaging the virus envelope and stopping the infection of HIV to T cells [8,21]. Some α-helical peptides such as Melittin and Cecropin have been reported to exert antiviral activities by direct disruption of virus membranes or inhibition of the virus replication [31]. The current study found that Caerin1.1 was able to destroy the structure of viral particles and reduce the viruses capable of infecting the cells and decrease their titers almost up to 3 logs. This is the first attempt to reveal that Caerin1.1 has strong antiviral activity against PEDV infection.

Some AMPs including Cecropin D (CD-PRRSV, SALPs-AIV) were reported to have blocked viral attachment and invasion by interacting with the receptors of the host cells [32]. Based on these findings, so we tried to keep PEDV infection within the periods of the attachment and entry through the temperature control, and the results showed no evident differences in protein expression among attachment period treated group, entry period treated group, and the PEDV control group. The number of virus infecting cells did not diminish. This means that Caerin1.1 did not interfere with the attachment and entry process. Considering the fact that Caerin1.1 does not compete with the virus to conjugate with the cells, and the fact that viral attachment-entry processes are very fast and Caerin1.1 will not have enough time to act against the viral membrane, it can be concluded that Caerin1.1 has less significant antiviral activities in the pre-incubation of Caerin1.1-cells progress. To further understand the mechanism in the post-treatment process, we examined the antiviral activity during the replication period. The results showed that Caerin1.1 did not have obvious antiviral effect during viral replication period, but it can control the infection progress by blocking the release of PEDV particles to reduce viral transmission among the adjacent cells.

Antiviral activities of Caerin1.1 is expected to be improved through the combination usages with other different AMPs or other antiviral agents like graphene oxide (GO), which could make Caerin1.1 a good weapon to deal with the diseases caused by viruses including PEDV [33]. As a matter of fact, we did a combination treatment with Piscidin which was also proved to be effective against PEDV [34], but the results were not as good as expected based on our unpublished work. The mechanisms still remain to be further investigated. Although the joint use of Piscidin and Caerin1.1 did not work as well as expected, other candidates can be explored for the prevention of PEDV and other pathogens in drug combination studies. On the other hand, some related research work has been done in our laboratory. Our other unpublished work has preliminarily testified the extraordinary ability of Caerin1.1 against some other viruses including PRV (Pseudorabies virus) which is a herpesvirus with double-stranded DNA that can infect many mammal species. Therefore, many potential functions of Caerin1.1 are still to be explored in the future.

## 5. Conclusions

In this study, we investigated the antiviral activities of Caerin1.1 against PEDV strains and its antiviral mechanism. The results show that Caerin1.1 can maintain the integrity of the host cells since it has very low cytotoxicity, and that it exhibits excellent virucidal activity in a dose-dependent manner even at very low concentrations. Caerin1.1 can reduce the viruses infecting the cells by destroying the integrity of the viral membrane, resulting in the decrease in the virus replication and protein expression. Caerin1.1 can also interfere with the virus release process essential for the inhibition of intracellular infection. Therefore, Caerin1.1 is an excellent antiviral material against PEDV. In conclusion, our study testifies the outstanding antiviral activities of Caerin1.1 and lays a foundation for future research and for the application of amphibian antimicrobial peptides.

## Abbreviations

AMP	Antimicrobial peptide
IFA	Indirect immunofluorescent assay
NMR	Nuclear magnetic resonance
MTT	3-(4,5-dimethyl-2-yl)-2,5-diphenyltetrazolium bromide
PED	Porcine epidemic diarrhea
PEDV	Porcine epidemic diarrhea virus
TCID_50_	Tissue Culture Infectious Dose 50
